# Coronavirus-related Health Literacy in elderly and adult population during COVID pandemic in Italy: Luigi Palmieri

**DOI:** 10.1093/eurpub/ckac130.135

**Published:** 2022-10-25

**Authors:** L Palmieri, A Rosano, C Lorini, C Cadeddu, G Bonaccorsi, B Unim, P De Castro, D Galeone, G Onder, C Donfrancesco

**Affiliations:** 1 Department of Cardiovascular Diseases, Istituto Superiore di Sanità, Rome, Italy; 2 National Institute for Public Policy Analysis, Rome, Italy; 3 Department of Health Sciences, University of Florence, Florence, Italy; 4 Department of Life Sciences and Public Health, Università Cattolica del Sacro Cuore, Rome, Italy; 5 Scientific Communication Unit, Istituto Superiore di Sanità, Rome, Italy; 6 Ministry of Health, Rome, Italy

## Abstract

**Background:**

The COVID-19 pandemic caused an overabundance of valid and invalid information rapidly spread via traditional media, by internet and digital communication. Health Literacy (HL), as the ability to access, understand, appraise, apply health information, is fundamental for finding, interpreting, correctly using COVID-19 information.

**Methods:**

In 2021, in the framework of the participation to the WHO M-POHL (Measuring Population and Organizational Health Literacy) network, a survey was conducted in a representative sample of the Italian general population aged 18+ years (N = 3,500). The validated HL questionnaire included coronavirus-related HL (HL-COVID, 16-items), general HL, sociodemographic characteristics, risk factors, lifestyles sections. For the HL-items, a 4-point Likert scale was applied: very easy, easy, difficult, very difficult. HL-COVID levels were defined as Good: very easy+easy>81.3% (more than 12 of 16 answers); Sufficient: 50.0%<very easy+easy < =81.3% (9-12 of 16 answers); Limited: very easy+easy < =50.0% (fewer than 9 of 16 answers). Elderly were responders aged 65+ years, the remaining ones were defined as adults. Ordinal Logistic Regression analysis was performed to assess the association of HL-COVID with sociodemographic characteristics (sex, age-group, educational level, financial deprivation).

**Results:**

Good HL-COVID prevalence was lower in elderly than in adults (44.8% vs. 51.0%, p-value=0.001); the opposite for both sufficient (22.8% vs. 19.9%) and limited (32.5% vs. 29.1%) levels, but not statistically significant. The odds of a low HL-COVID (sufficient/limited) increased by 31% in the elderly and by 50%, 92%, and almost triple in persons with a low, considerable, and severe financial deprivation level, respectively.

**Conclusions:**

The COVID-19 pandemic highlighted the need to improve HL and to prepare the general population for future emergency and non-emergency situations, confirming that HL can be considered a social vaccine.

**Key messages:**

- Low coronavirus-related HL level is mostly associated to elderly and to increasing deprivation level.
- The COVID-19 pandemic confirmed that Health Literacy can be considered a social vaccine.

